# Introduction of *Panax notoginseng* into pine forests significantly enhances the diversity, stochastic processes, and network complexity of nitrogen-fixing bacteria in the soil

**DOI:** 10.3389/fmicb.2025.1531875

**Published:** 2025-02-03

**Authors:** Xiaoyan Zhao, Shu He, Rui Rui, Jingying Hei, Xiahong He, Shu Wang

**Affiliations:** ^1^Yunnan Provincial Key Laboratory for Conservation and Utilization of In-forest Resource, Southwest Forestry University, Kunming, China; ^2^Southwest Research Center for Engineering Technology of Landscape Architecture (State Forestry and Grassland Administration), Kunming, Yunnan, China

**Keywords:** *Panax notoginseng*, nitrogen-fixing bacteria, *nifH* gene, high-throughput sequencing, structural equation model

## Abstract

**Introduction:**

Nitrogen-fixing bacteria (NFB) have a pivotal impact on the nitrogen cycle within agroforestry systems. The organic management of the *Panax notoginseng* (sanqi)-*Pinus armandii* agroforestry (SPA) system resulted in nitrogen deficiency because of the lack of application of chemical fertilizers. Therefore, assessing the variability in NFB due to the cultivation of sanqi in the SPA system becomes crucial.

**Methods:**

The seasonal dynamics in the abundance, diversity, and community structure of NFB in the soil of monocropping pine (MP) and SPA systems were assessed using real-time quantitative polymerase chain reaction and high-throughput sequencing technology.

**Results and discussion:**

Sanqi cultivation triggered a decrease in the abundance of NFB but increased *α* diversity. Additionally, significant differences in the community structure of NFB were noted between the MP and SPA systems. Moreover, the abundance of *Bradyrhizobium* and *Azospirillum* increased in the soil after sanqi was cultivated. Furthermore, the cultivation of sanqi broadened the ecological niche breadth of NFB and increased the stochasticity in its community structure assembly (i.e., dispersal limitation). Additionally, the SPA system increased the network complexity but not the stability of NFB. The structural equation model (SEM) revealed that pH directly impacted the network complexity and stability of NFB in the SPA system. Sanqi cultivation positively influences the community characteristics of NFB in the soil in the SPA system. Our study provides new insights into nitrogen cycling and utilization in the SPA system.

## Graphical abstract

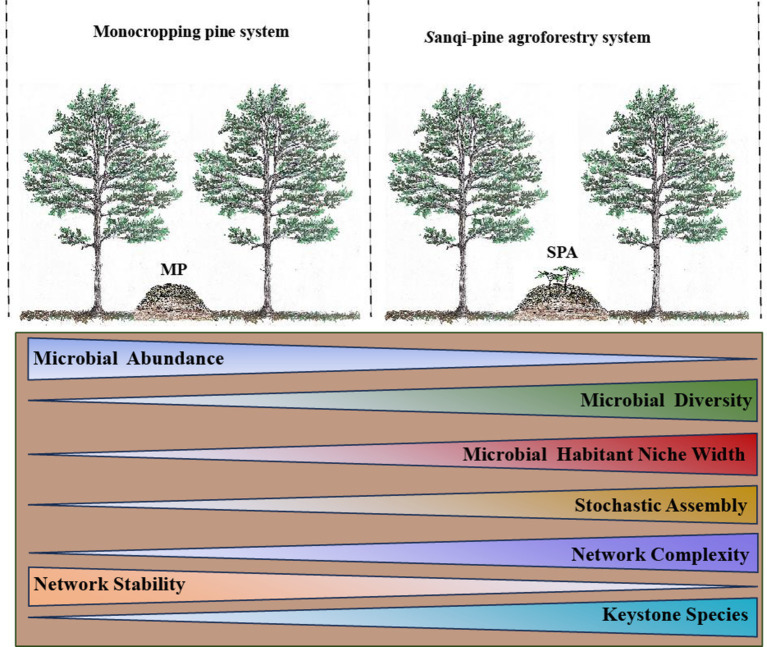

## Highlights

- Sanqi cultivation triggered a decrease in the abundance of nitrogen-fixing bacteria.
- Sanqi cultivation increased the assembly stochasticity of nitrogen-fixing bacteria.
- The network complexity but not the stability of nitrogen-fixing bacteria was increased following Sanqi planting.
- pH directly impacted the network complexity and stability of nitrogen-fixing bacteria.

## 1 Introduction

Biological nitrogen fixation is an essential step of the nitrogen cycle (Levy-Booth et al., 2014) as it is the largest source of biologically-available nitrogen (Canfield et al., 2010) and the biggest contributor to the global reservoir of nitrogen of most terrestrial ecosystems (Galloway et al., 2004). Nitrogen-fixing bacteria (NFB) utilize nitrogenase to reduce atmospheric gaseous nitrogen (N_2_) to ammonia that is easily absorbed and utilized by plants (Rilling et al., 2018). The *nifH* gene encodes the ferritin subunit of nitrogenase, it is therefore essential for nitrogen fixation (Ininbergs et al., 2011) and serves as a marker gene for NFB in terrestrial ecosystems (Chen et al., 2019). Agriculture, forest, and agroforestry systems have the potential to annually fix 40–70, 55, and 246.4 Tg N (Cleveland et al., 1999; Vitousek et al., 2013; Kim and Isaac, 2022), respectively, via the activity of NFB. The elevated rate of nitrogen fixation in the agroforestry system is attributed to the enhanced utilization of nitrogen owing to the introduction of plants, which subsequently affects NFB (Dang et al., 2024). Therefore, the alterations in the abundance and community structure of NFB can be utilized as a reliable indicator for evaluating nitrogen cycling in different agroforestry systems (Wang S. S. et al., 2023) and reflect the status of nitrogen utilization in these systems (Zhong et al., 2022).

An agroforestry system combines trees and crops within the same land unit (Kwak et al., 2019), which notably impacts the NFB community (Solanki et al., 2017). Due to the introduction of different plant species, the abundance and diversity of NFB in the soil varied across the agroforestry systems. For instance, some agroforestry systems such as the red oak–soybean (Graungaard, 2015), poplar–wheat/sweet potato (Kong et al., 2015), and *Hippophae rhamnoides–Pinus tabuliformis/Platycladus orientalis*/*Robinia pseudoacacia* (Yang et al., 2015) systems can significantly enhance the abundance of NFB. By contrast, other agroforestry systems such as *Macadamia ternifolia*/*Populus euphratica–Hordeum vulgare* (Beule and Karlovsky, 2021; Wang R. L. et al., 2023) can either reduce or not exert any effect on the abundance of NFB. Moreover, the sugarcane–peanut/soybean agroforestry system remarkably enriched the diversity of NFB (Liu et al., 2021; Pang et al., 2022). Mulberry–alfalfa (Zhang et al., 2018), citrus–poplar (Gopal et al., 2021), tea–mung bean/adzuki bean (Wang D. L. et al., 2023), and tea–soybean/canola (Zhong et al., 2022) agroforestry systems notably altered the community composition of NFB concomitantly with an increase in the abundance of Proteobacteria, *Rhizobium*, and *Burkholderia*. These different findings can be attributable to alterations in environmental factors among the agroforestry systems, including soil metabolites (Qiao et al., 2024), edaphic factors (Kerfahi et al., 2016), plant species (Arafat et al., 2017), microbiome (Hayden et al., 2010), and enzyme activity (Xie et al., 2022). Additionally, seasonal dynamics had a notable influence on the abundance and diversity of NFB (Li H. P. et al., 2022; Zhang et al., 2021). The seasonal dynamics directly/indirectly impact the communities of NFB in the soil by regulating energy influx, carbon source availability, and carbon source quality via variations in temperature and moisture (Pereira et al., 2013; Che et al., 2018). Briefly, a comprehensive assessment of the abundance and community structure of NFB in agroforestry systems and of the influencing factors establishes a theoretical foundation for improving nitrogen utilization through improved management practices in agroforestry systems.

Co-occurrence networks are critical for understanding the community structure of NFB, as they offer new insights beyond diversity and community composition, elucidating intricate interactions among community members (Wu et al., 2019; Zheng et al., 2021). It has been established that plant introduction positively impacts the network properties of NFB in soil. For instance, a study on *Alternanthera philoxeroides* showed that demonstrated that its introduction substantially enhanced the network complexity and stability of NFB in soil (Li C. C. et al., 2022), potentially due to heightened nutrient effectiveness (Cao et al., 2020) and ecological niche differentiation (Zhang and Okabe, 2020). Furthermore, investigations into community assembly mechanisms can illuminate the shaping process of microbial communities (Zhou and Ning, 2017). It is widely acknowledged that both stochasticity (neutral theory) and determinism (ecological niche theory) jointly shape the community structure of NFB (Zhou et al., 2014), yet this balance can be affected by external factors such as pH (Fan et al., 2018b), fertilizer application (Feng et al., 2018), altitude (Wang et al., 2019), etc. However, the specific impact of Sanqi introduction on the co-occurrence network of NFB in SPA system and its underlying assembly mechanisms remain unclear.

*Panax notoginseng* (sanqi), mainly found in the Yunnan and Guangxi Provinces (Hei et al., 2024), is a precious perennial medicinal herb known for its antitumor (Leung et al., 2007), immunity–boosting (Liu et al., 1995), anti–inflammatory (Jiao D. L. et al., 2022), and blood pressure–lowering (Loh et al., 2019) effects. Compared to conventionally managed sanqi, the cultivation of sanqi under the forest understory in the Yunnan Province has been widely popularized (Hei et al., 2023) owing to the advantages it offers with respect to the alleviation of continuous cropping obstacles (Rui et al., 2024), quality enhancement of sanqi (Li et al., 2024), and the delivery of beneficial microorganisms for pine tree growth (Jia et al., 2022). The introduction of plants into agroforestry systems results in the consumption of a large quantity of nitrogen (Wakelin et al., 2010). However, the organic management of the sanqi-pine agroforestry (SPA) system without the application of chemical pesticides and fertilizers resulted in nitrogen deprivation. Moreover, the subtropical forests in the Yunnan Province, especially the monoculture pine (MP) forests, are subjected to significant nitrogen limitation (Luo et al., 2020). Presently, the impact of converting the MP forests to SPA systems on the community characteristics of the NFB remains uncertain. Therefore, we established MP and SPA systems and analyzed the seasonal dynamics in the abundance and community of NFB using real-time quantitative polymerase chain reaction and high-throughput sequencing technology. Furthermore, we conducted an analysis on the association between NFB and edaphic factors. The research objectives of the current study include the following: (1) exploring the effects of sanqi cultivation on the abundance, diversity, and community structure of NFB, and (2) exploring the main factors that affect the NFB.

## 2 Materials and methods

### 2.1 Study area and soil collection

The study site includes sanqi from the forest understory base located in Xundian County, Kunming (China) at an elevation of 2,199 meters (103°12′45″ E, 25°28′18″ N). The region experiences a monsoon climate, with an annual rainfall and average temperature of 1,900 mm and 14.5°C, respectively. *Pinus armandii*, the main species of tree that is predominantly found in this region, has a lifespan of 30 years, a canopy density ranging from 0.7 to 0.9, an average trunk diameter of 18 cm, and a height of 9.5 m. The plant-row spacing of sanqi planted in *P. armandii* forests is maintained at 10 to 15 cm by 10 to 15 cm. Furthermore, the planting density of sanqi in these pine forests is set at 14,000 plants per 667 m^2^. The method of cultivation and daily management practices for the sanqi from the forest understory were based on the findings reported by Hei et al. (2024).

Tillage and ridging were carried out in the *P. armandii* forest followed by the establishment of control (MP) and treatment (SPA) systems without and with sanqi plantation, respectively (Supplementary Figure S1). Each treatment consisted of three 20 m × 20 m plots, totaling six plots. The soil samples were collected on the 10^th^ and 20^th^ of each month from September 2020 to August 2021, resulting in a total of 144 samples (6 plots × 12 months × 2 times/month). All soil samples were meticulously analyzed for their physicochemical properties and the abundance of NFB. Furthermore, we collected soil samples across four distinct sampling periods—10th October 2020 (autumn), 10th January 2021 (winter), 10th April 2021 (spring), and 10th July 2021 (summer)—for high-throughput sequencing analysis. Five soil cores, each ranging from 0 to 20 cm in depth, were collected from each plot utilizing the five-point sampling technique and then blended together to create a composite sample (Jia et al., 2024). Aliquots of the soil samples were rapidly transferred to the laboratory and stored at −4°C and − 80°C for subsequent analysis.

### 2.2 Analysis of edaphic factors

Soil temperature (ST) was determined at a depth of 0–20 cm with a chromium-plated soil thermometer (WNG-11, Tianjin Jixing Instrument Factory, China). Soil moisture (SM) was calculated by the difference between the wet and dry weights of the soil, while soil bulk density (BD) and water-filled pore space (WFPS) were obtained using the ring-knife technique. Soil pH was determined with a pH meter (AB23 PH-F, OHAUS, United States). The continuous flow analyzer (Auto Analyzer AA3, Seal, Germany) was used for determining the content of ammonium nitrogen (NH_4_^+^–N), nitrate nitrogen (NO_3_^−^–N), total nitrogen (TN), and total phosphorus (TP). The soil was treated with hydrofluoric and perchloric acids for digestion, followed by the determination of total potassium (TK) using atomic emission spectroscopy with an AA-6300C flame photometer (SHIMADZU, Japan). The potassium dichromate oxidation method was used for estimating soil organic carbon (SOC).

### 2.3 qPCR analysis

DNA from the soil microbes was extracted from fresh soil samples (0.5 g) using the Fast DNA® SPIN Kit (MP Biomedical, United States) as per the manufacturer’s protocol. The concentration and quality of the isolated DNA were estimated according to standard methods (Jia et al., 2024). The quantification of NFB was conducted using the LightCycler® 480 II System (Roche, Basel, Switzerland). The primer sequences and reaction conditions are specified in Supplementary Table S1. The target product containing *nifH* gene was isolated with a plasmid extraction kit (Takara), and its concentration was determined. The plasmid was then subjected to a tenfold serial dilution (10^−1^–10^−7^) to construct a standard curve with an amplification efficiency of 93.94% and a slope of −3.48. The initial copy number in the samples was established by comparing the Cp values of the amplified samples to that of the standard curve; the experiment was repeated thrice for each sample.

### 2.4 DNA extraction and sequencing

The procedure for DNA extraction and the primer sequences were consistent with those used for qPCR analysis, and the amplification conditions are recorded in Supplementary Table S1. After purification and quantification with the Gel Recovery Kit (Axygen Biosciences, USA) and QuantiFluor TMST (Promega, United States), respectively, the purified products were combined in an equimolar ratio as per the protocol provided and subsequently subjected to sequencing analysis.

Following the removal of the primers and barcode sequences, the raw data were subjected to quality control and assembly with fastp v.0.20.0 and FLASH v.1.2.11 software (Magoč and Salzberg, 2011; Chen et al., 2018), respectively. The sequences were grouped into operational taxonomic units (OTUs) at a similarity threshold of 97% using UPARSE 7.1 software, followed by the removal of chimeras (Edgar, 2013). Subsequently, species classification for the sequences was performed in the *nifH* database with the RDP v 2.13 classifier (Gaby and Buckley, 2014), with the exclusion of OTUs from chloroplasts and mitochondria. The “VEGAN” package was utilized for the standardization of all samples (Dixon, 2003). The original sequence was deposited to the sequence read archive database (accession number: PRJNA1173392).

### 2.5 Statistical analysis

Pairwise Spearman’s correlation analysis was performed on the OTUs using the “psych” package, OTUs with |R| > 0.6 and *p* < 0.05 were retained (Zheng et al., 2021). The visualization of the co-occurrence network was conducted using Gephi v 0.9.2 software (Bastian et al., 2009). The approach suggested by Xu et al. (2023) was followed to evaluate the network complexity and stability (Wu et al., 2023). The critical nodes in the network (with Zi > 2.5 or Pi >0.62) were identified as their removal may lead to network collapse (Yuan et al., 2021).

The Chao1 and Shannon’s indices was calculated with Mothur v1.30.2 software. The “vegan” and “ggplot2” packages were employed to generate PCoA plots and stacked bar graphs for visualizing community structure and composition, respectively. SPSS 19.0 (IBM, Armonk, NY, United States) was used for statistical analysis of soil characteristics, *nifH* gene abundance, *α* diversity (including Chao1 and Shannon index), and the relative abundance of major genera of NFB. To investigate the effect of soil properties on the community structure of NFB, we used the top 10 genera and soil physicochemical properties for redundancy analysis (RDA). The ecological niche breadth of the NFB was determined using the methodology detailed in Li et al. (2021). The neutral (Sloan et al., 2006) and null (RC_Bray_ index, Ning et al., 2020) models were utilized for inferring the mechanisms underlying the variation in community structure. The collinear clustering analysis of soil physical and chemical factors was carried out, and 9 key factors were found out from 11 factors. Random forest algorithms were performed using the packages “rfPermute” (Archer, 2016) and “A3” (Fortmann-Roe, 2015) to assess the variables (key factors) of network stability and model significance, respectively. The nutrient elements included TK, NH_4_^+^-N, and NO_3_^−^-N. Network complexity included node number and edge number, average degree, average path length, graph diameter, graph density and clustering coefficient. Network stability refers to the absolute value of negative/positive cohesion. Structural equation model (SEM) was constructed using Amos 28.0 (AMOS IBM) to determine the effects of pH, nutrient elements, α diversity, and network complexity on network stability. The model’s reliability was evaluated using the R^2^-value, Chi-square test, degrees of freedom, and *p*-value, root-mean-square error of approximation (RMSEA), and goodness-of-fit index (GFI).

## 3 Results

### 3.1 Edaphic properties of the soil

The average edaphic factors in the MP and SPA systems over the year was listed in Supplementary Table S2 and Figure S2. The ST, SM, BD, TN and WFPS as well as the contents of TK, NO_3_^−^–N, and SOC were significantly elevated in the soil of the SPA system compared to that of the MP system, while the pH and contents of TP, and NH_4_^+^–N were noticeably reduced (*p* < 0.05; Supplementary Table S2). In the MP system, the SM, WFPS, and the contents of TP and NH_4_^+^–N were the highest during autumn and lowest during spring (Supplementary Table S3). In the SPA system, soil pH and the contents of TN and NO_3_^−^–N were the highest during spring and lowest during summer (Supplementary Table S3).

### 3.2 Copy number of the *nifH* gene

**Figure 1:**
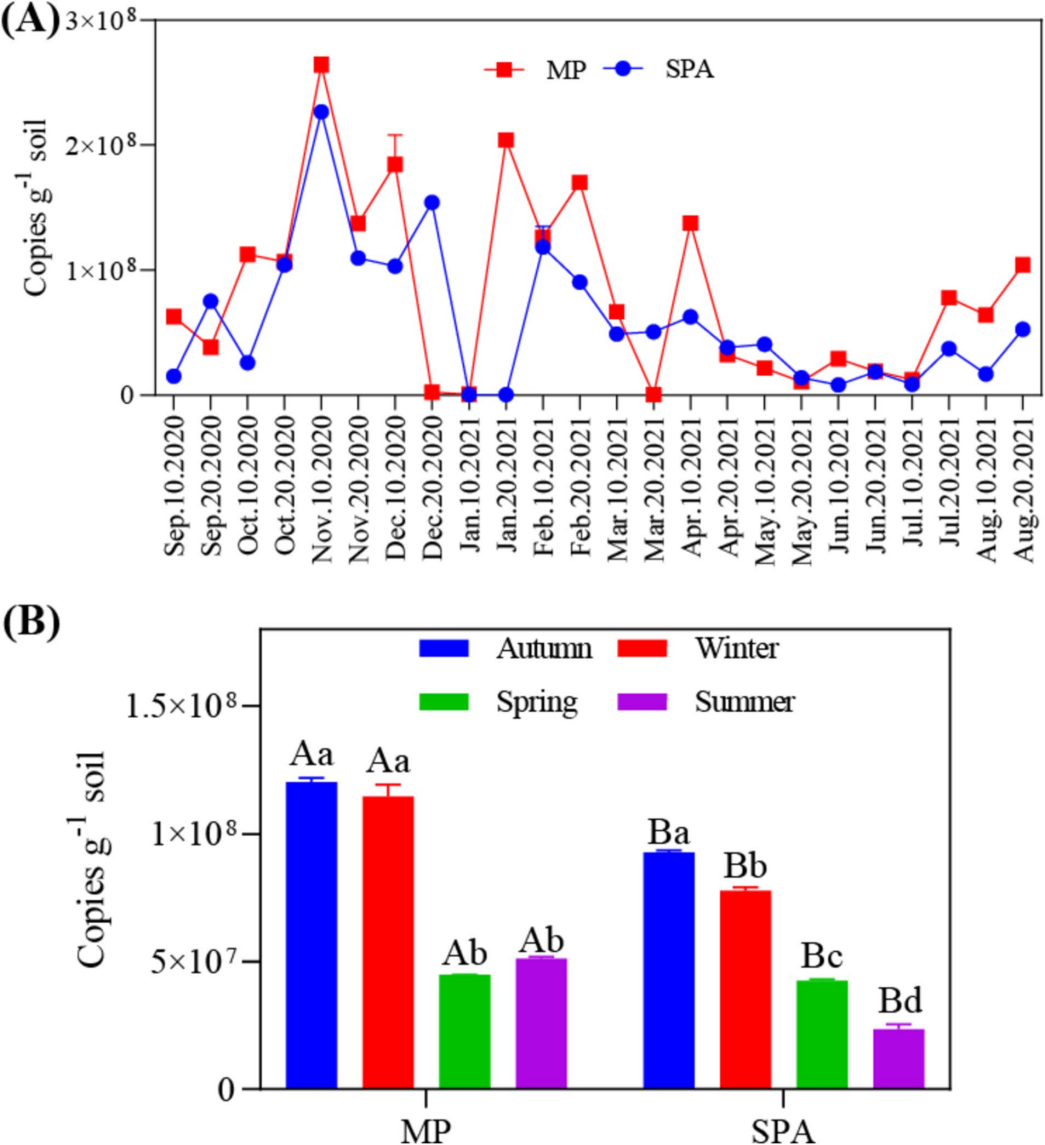
Annual **(A)** and seasonal **(B)** variations in the abundance of NFB in the soil.

The data collected over the year revealed that the abundance of the *nifH* gene ranged from 4.22 × 10^5^ to 2.70 × 10^8^ and 4.03 × 10^5^ to 2.28 × 10^8^ copies g^−1^ soil in the MP and SPA systems, respectively (Figure 1A). The soil from the MP system exhibited significantly higher abundance of *nifH* than that of the SPA system (*p* < 0.01), with peak abundance of *nifH* recorded during autumn and winter (Figure 1B). Correlation analysis indicated that ST and TP were the essential factors that exhibited negative and positive correlation, respectively, with the abundance of NFB (*p* < 0.05; Table 1).

**Table 1:** Correlation between the abundance of NFB and edaphic factors over a year duration.

	ST	SM	pH	BD	TK	TP	NH_4_^+^–N	NO_3_^−^–N	SOC
Abundance^all-round year^	−0.359**	0.061	−0.012	−0.09	−0.057	0.187*	−0.13	0.057	0.057
Abundance^MP^	−0.314**	0.014	−0.22	0.036	−0.098	0.168	−0.299*	0.260*	0.195
Abundance^SPA^	−0.435**	0.448**	0.008	−0.05	0.191	0.311**	−0.013	0.156	−0.143

“*” and “**” indicate statistically significant differences at the *p* <0.05, and *p* < 0.01 levels, respectively.

### 3.3 Analysis of diversity and community structure of NFB

**Figure 2:**
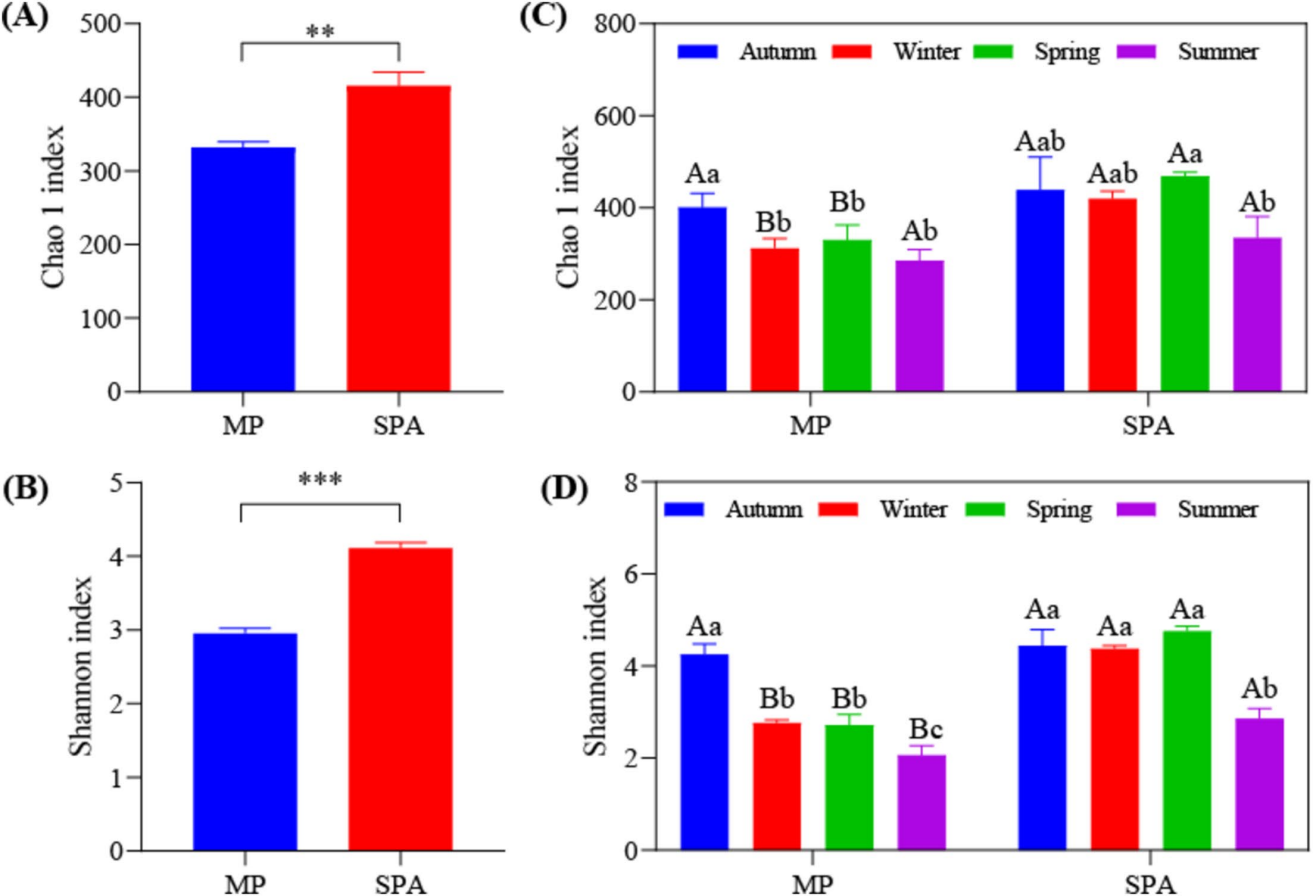
Analysis of α diversity of NFB in MP and SPA systems. Average of Chao1 **(A)** and Shannon **(B)** index; Seasonal changes of Chao1 **(C)** and Shannon **(D)** index.

High-throughput sequencing was carried out using the microbial DNA isolated from soil sampled from the two systems in October 10th 2020, January 10th 2021, April 10th 2021, and July 10th 2021. The dilution curve demonstrated that the sequencing depth adequately encompassed most species of the *nifH* microbial community (Supplementary Figure S3). The *α* diversity (Chao1 and Shannon’s indices) was notably elevated in the SPA system compared to the MP system (*p* < 0.01; Figures 2A,B) and was lowest in summer (Figures 2C,D). Correlation analysis revealed that SM and the contents of TK, TP, and NH_4_^+^–N were the crucial factors regulating the α diversity of NFB (Table 2).

**Table 2:** Association between edaphic factors and α diversity of NFB.

	ST	SM	pH	BD	TK	TP	NH_4_^+^–N	NO_3_^−^–N	SOC
Chao1^all^	−0.093	0.411*	−0.195	0.282	0.467*	0.451*	−0.463*	0.078	0.337
Shannon^all^	−0.205	0.454*	−0.259	0.376	0.556**	0.580**	−0.426*	0.161	0.321
Chao1^MP^	0.034	0.253	−0.665*	0.299	0.563	0.786**	−0.265	0.795**	0.343
Shannon^MP^	−0.086	0.332	−0.756**	0.495	0.673*	0.916**	−0.158	0.801**	0.239
Chao1^SPA^	−0.324	0.740**	0.647*	−0.071	−0.501	0.511	−0.649*	0.504	0.18
Shannon^SPA^	−0.541	0.751**	0.764**	0.027	−0.458	0.653*	−0.773**	0.665*	0.323

“*” and “**” indicate statistically significant differences at the *p* <0.05, and *p* < 0.01 levels, respectively.

**Figure 3:**
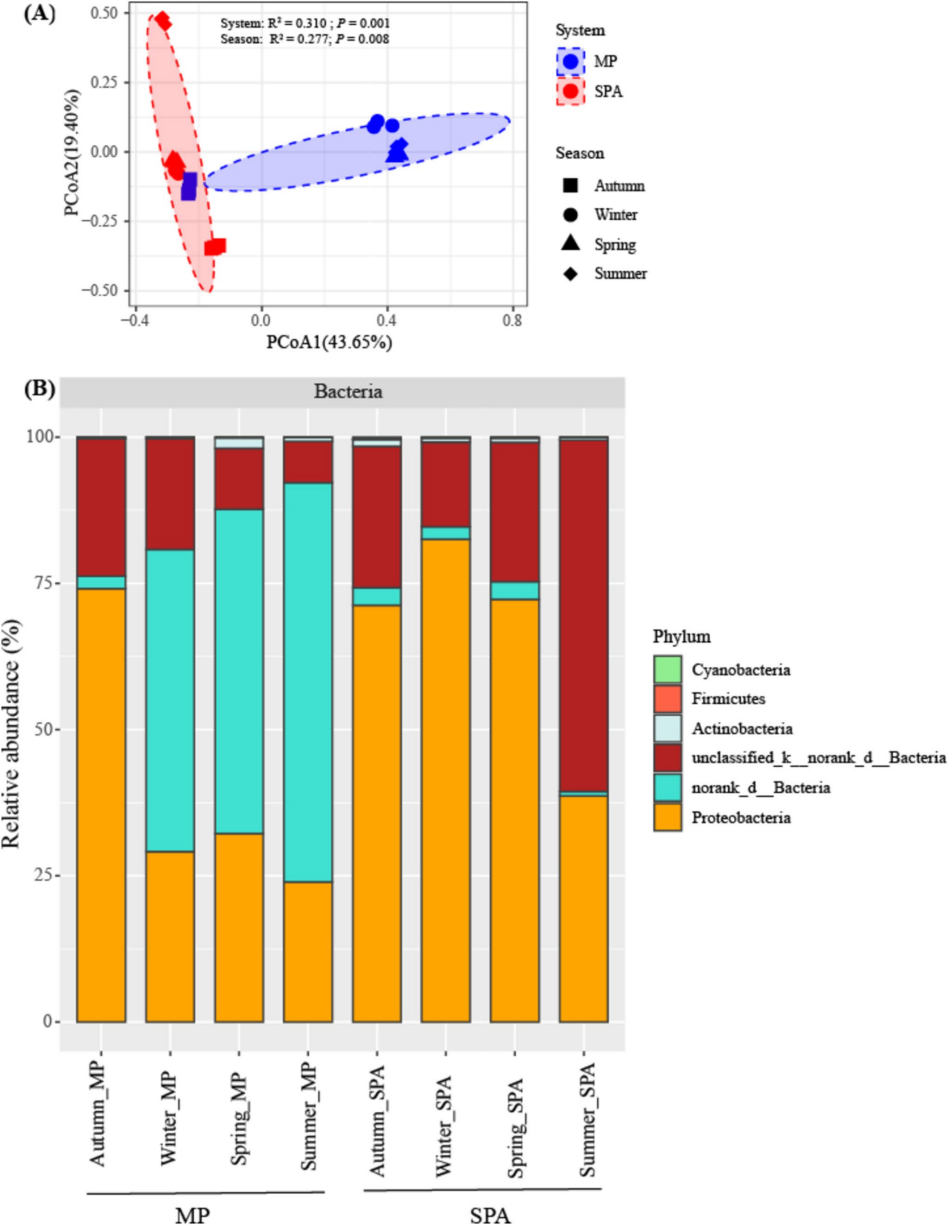
Analysis of the community structure **(A)**, and composition **(B)** of the NFB at a phylum level.

The components PCoA1 and PCoA2 of the PCoA contributed to 43.65 and 19.40%, respectively, of the variability within the community structure of NFB. This finding suggests a substantial distinction between the two systems with respect to the component PCoA1. Permutation multivariate analysis of variance indicated that system exerted a more pronounced influence on community structure (R^2^ = 0.310, *p* < 0.01), followed by the season (R^2^ = 0.277, *p* < 0.01, Figure 3A; Supplementary Table S4). Redundancy analysis (RDA) demonstrated that SM, TP and NH_4_^+^–N were essential factors that shaped the community structure of NFB (*p* < 0.01; Supplementary Figure S4).

The community composition of NFB was subsequently analyzed at the levels of phylum and genus. Six NFB phyla were identified, with the combined abundance of the top three dominant phyla (norank_d__Bacteria, unclassified_k__norank_d__Bacteria, and Proteobacteria) ranging from 98.08 to 99.70% (Figure 3B). Moreover, the relative abundance of five bacterial genera, namely *Bradyrhizobium*, *Azospirillum*, *Anaeromyxobacter*, *Beijerinckia*, and *Xanthobacter*, was dramatically higher in the SPA system compared to the MP system. By contrast, norank_d__Bacteria exhibited an opposite trend. *Paraburkholderia*, *Frankia*, and *Sphingomonas* did not display substantial variations between the two systems (Supplementary Table S5).

### 3.4 Evaluation of NFB assembly

**Figure 4:**
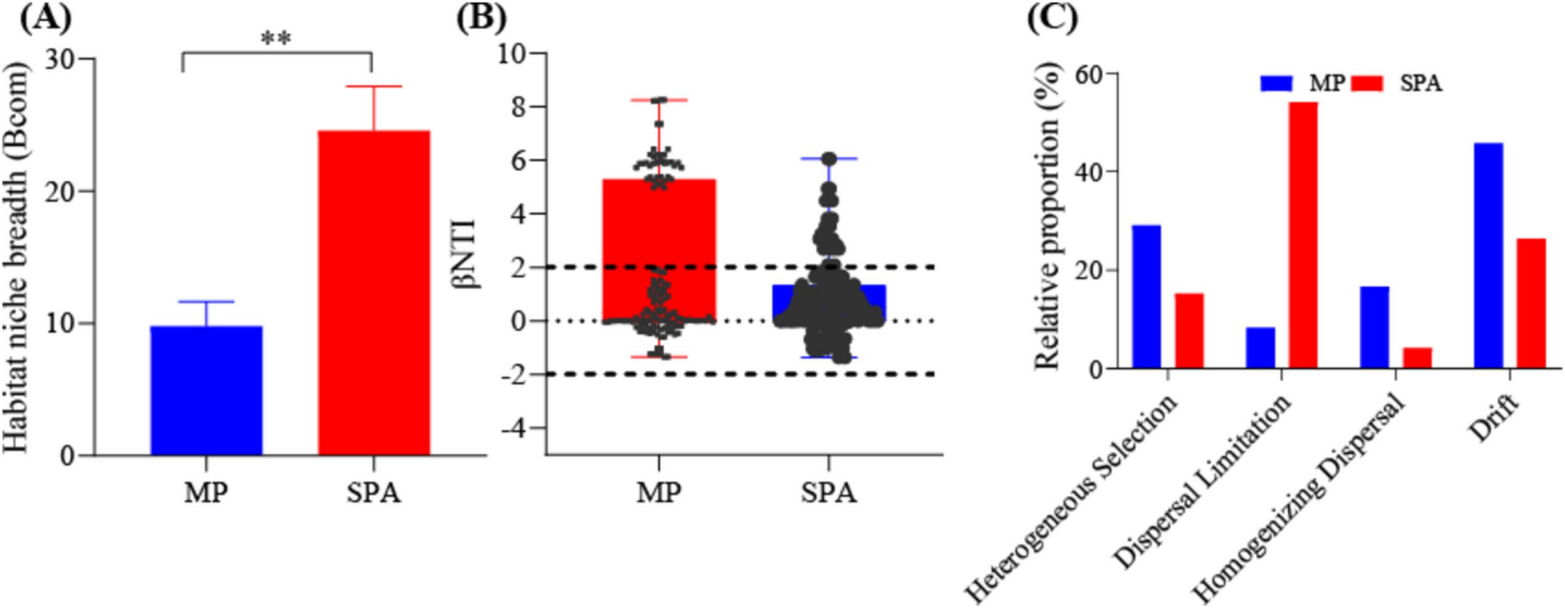
Analysis of the ecological niche breadth **(A)**, βNTI index **(B)**, and null-model **(C)** of NFB in the MP and SPA systems.

The adaptability of the NFB to the environment was assessed by calculating the ecological niche breadth in the two systems. The results indicated significantly greater ecological niche breadth for the NFB in the SPA system compared to those in the MP system (*p* < 0.05, Figure 4A). Moreover, the neutral model accounted for 27.5 and 36.6% of the variation of NFB in the MP and SPA systems, respectively, suggesting that the cultivation of sanqi increased the assembly stochasticity of the community structure of NFB (Supplementary Figures S5A,B). The βNTI and RC_Bray_ indices further confirmed that drift and dispersal limitation were the main factors that shaped NFB communities in the MP and SPA systems, respectively (Figures 4B,C). In conclusion, the cultivation of sanqi significantly impacts the community structure of NFB.

### 3.5 Co-occurrence network analysis of NFB communities

**Figure 5:**
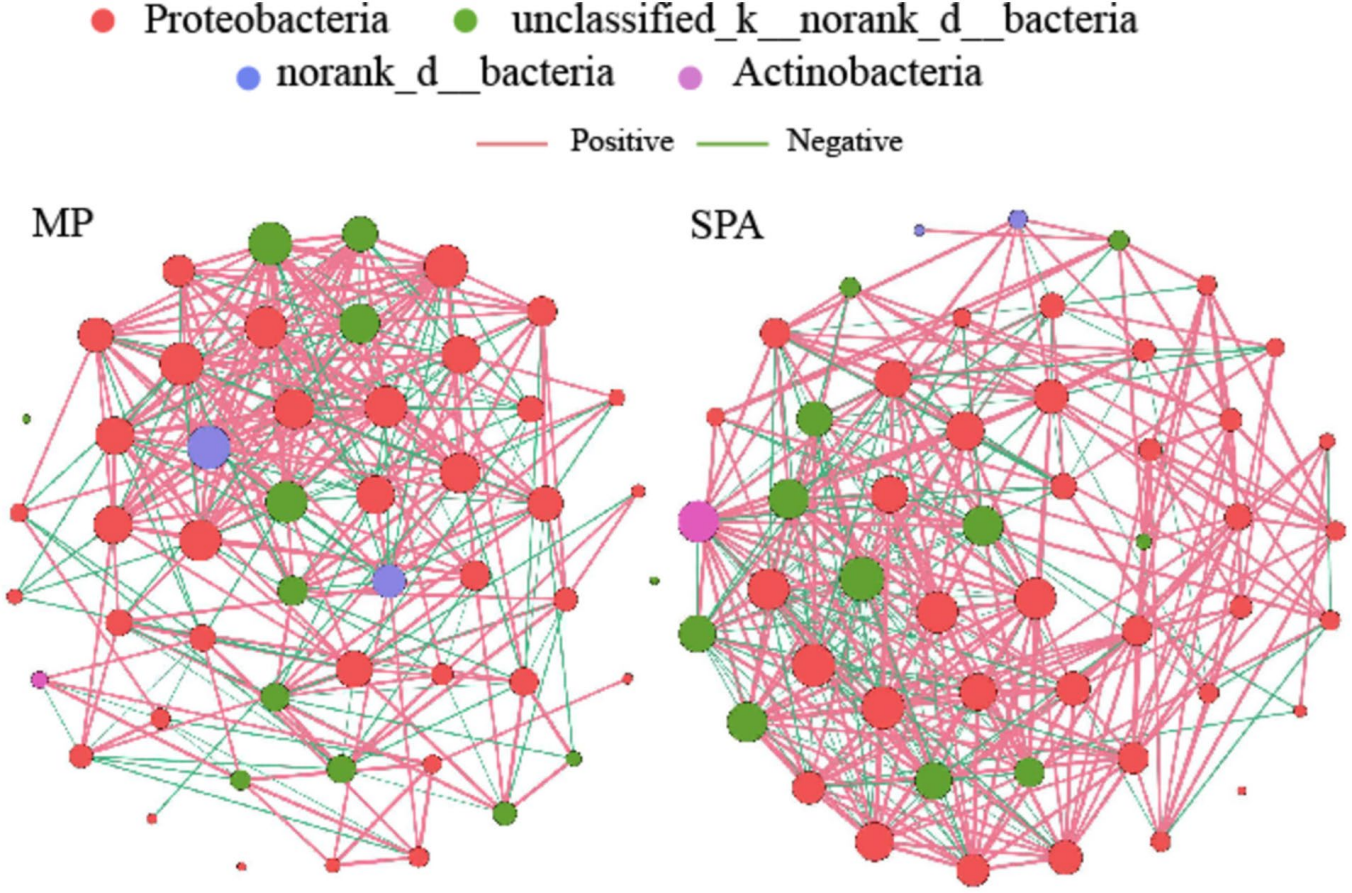
Co-occurrence networks of soil NFB communities in MP and SPA systems. Red and green lines represent positive and negative correlations, respectively.

**Figure 6:**
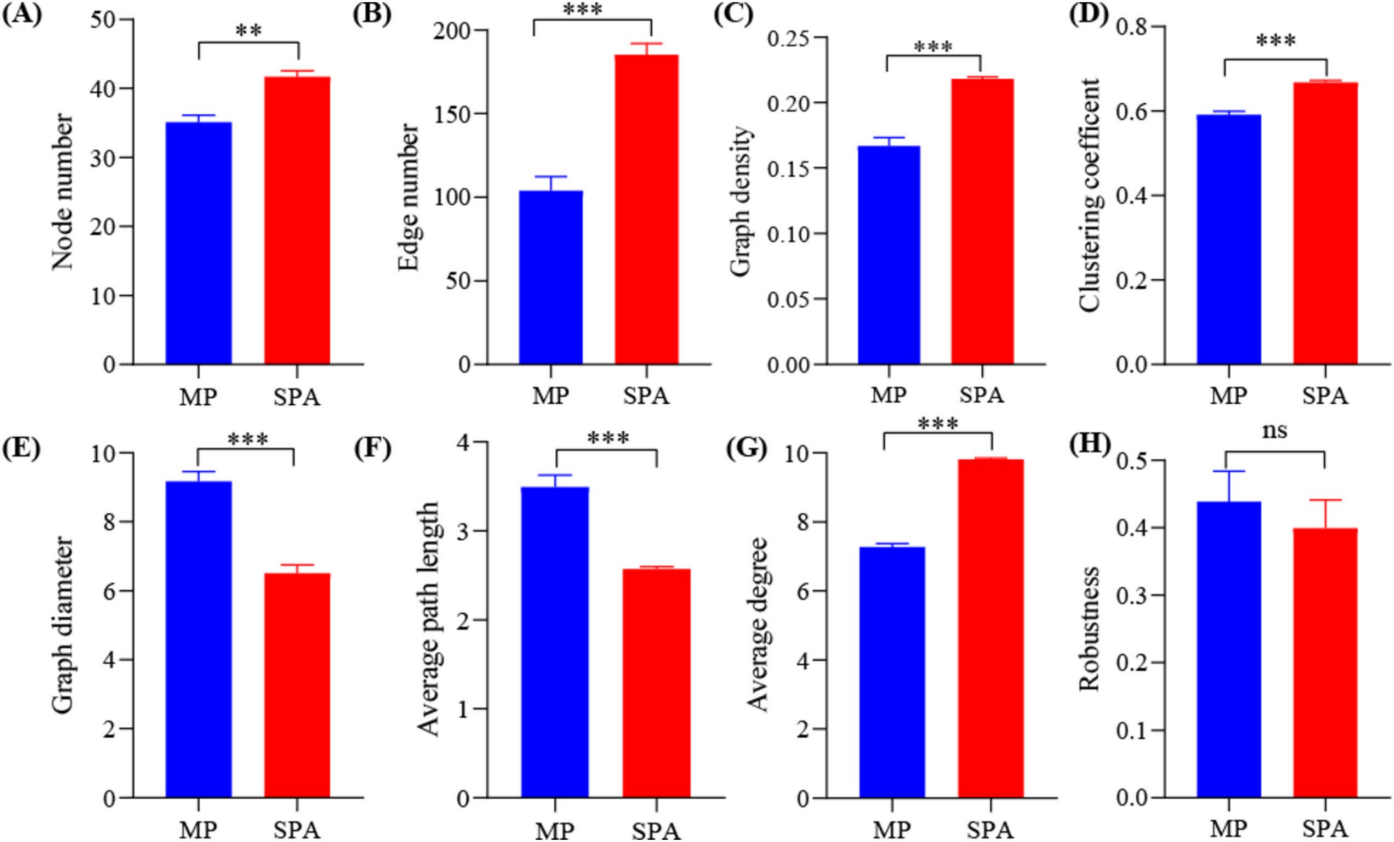
Network topology **(A–G)** and robustness **(H)** in MP and SPA systems.

A NFB network based on the top 50 OTUs was constructed to describe the associations within the communities of both MP and SPA systems (Figure 5). The network complexity of NFB was found to be greater in the SPA system compared to the MP system (*p* < 0.05; Figures 6A–G), with no substantial impact on network stability (Figure 6H).

**Figure 7:**
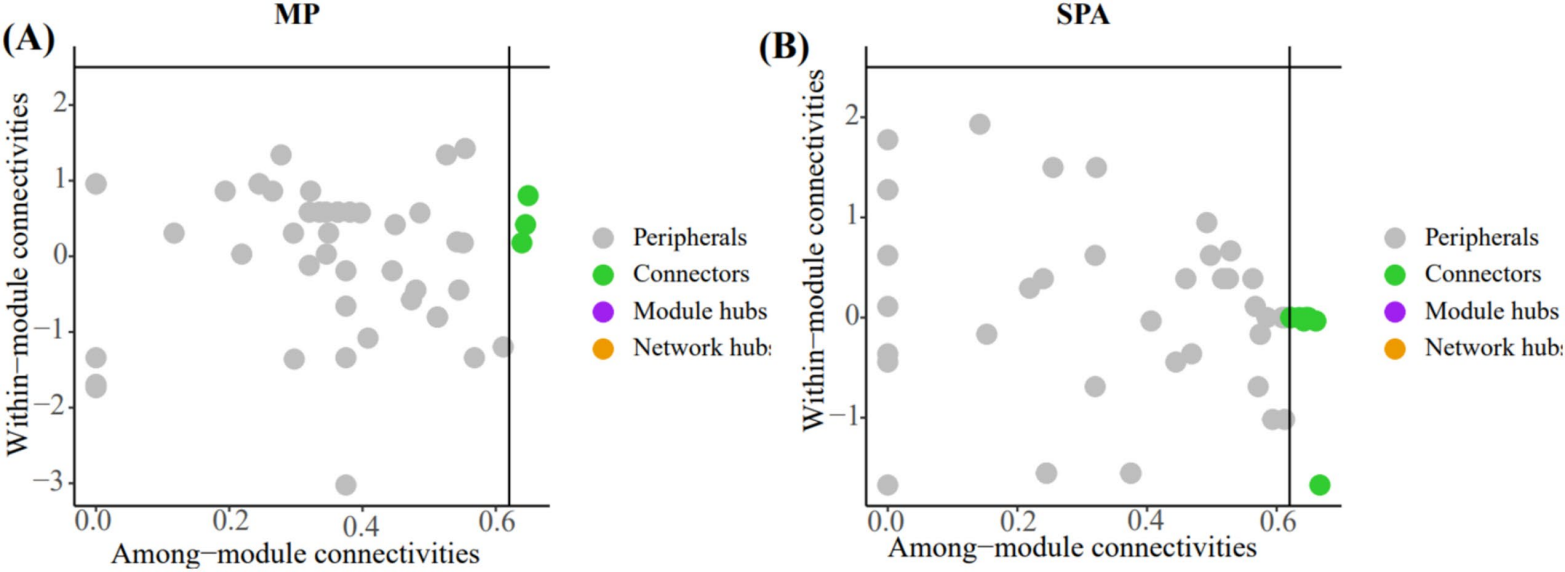
In analyzing the key OTUs in the soil NFB network of MP system **(A)** and SPA system **(B)** using the Zi-Pi method, the nodes (OTUs) in the green solid circles are defined as connectors.

The crucial nodes (OTU) in the network were determined through analysis of within-module (Zi) and between-module (Pi) interactions. Eleven connectors were detected in the two systems (Figures 7A,B; Supplementary Table S6). The three connectors (OTU1217, OTU1227, and OTU584) in the MP system were annotated as Rhizobiales, unclassified_k__norank_d__Bacteria, and norank_d__Bacteria, respectively. Additionally, the eight connectors (OTU1312, OTU1332, OTU1373, OTU510, OTU512, OTU532, OTU599, and OTU766) in the SPA system were assigned to the Rhizobiales (4), Burkholderiales (1), unclassified_p__Proteobacteria (2), and unclassified_k__norank_d__Bacteria (1), respectively.

### 3.6 SEM analysis

**Figure 8:**
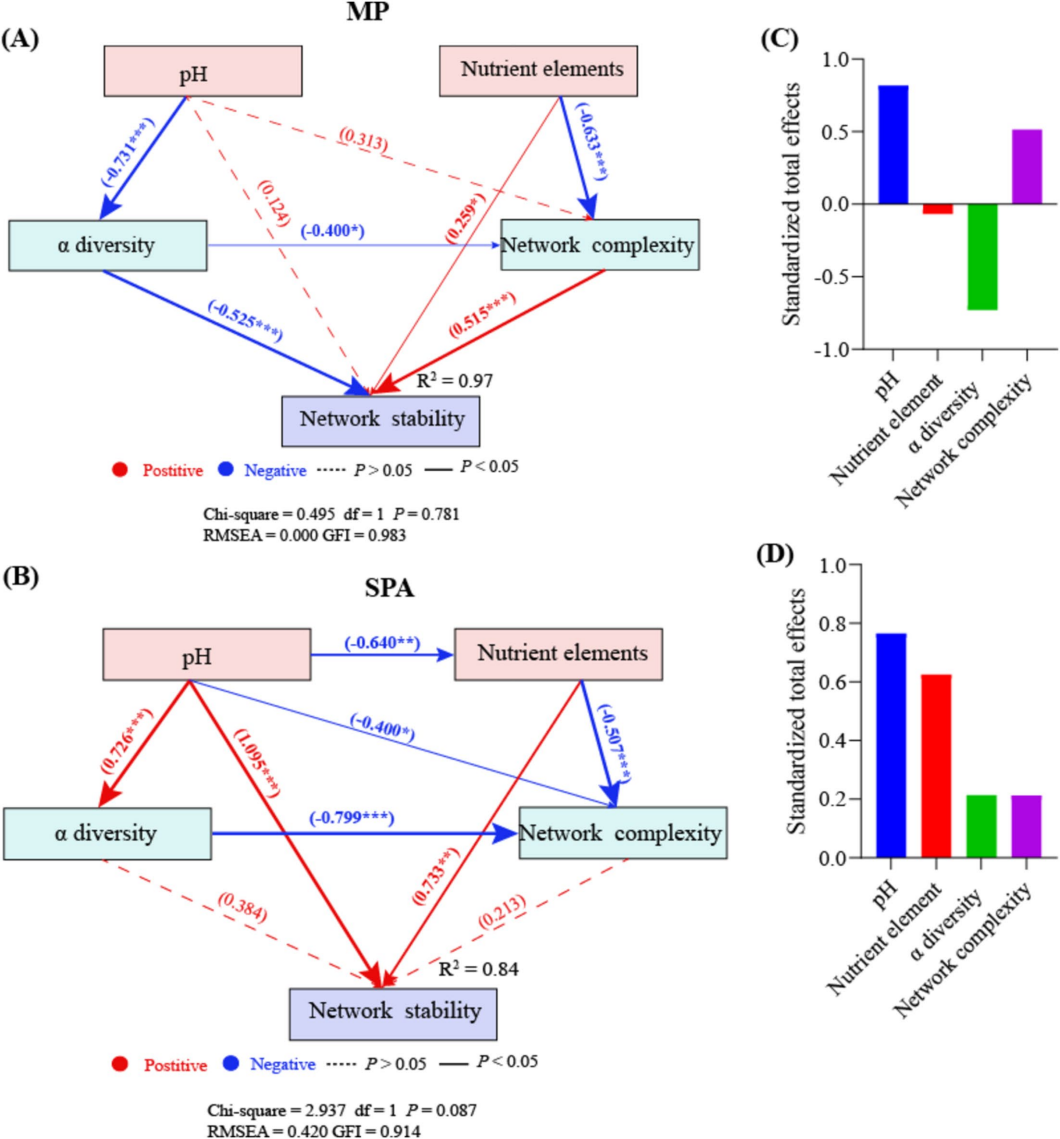
SEM analysis of the direct and indirect effects on the network stability of NFB in MP **(A)** and SPA **(B)** systems. The standardized total effects of each indicator in the SEM of the MP **(C)** and the SPA system **(D)**.

Random forest analysis indicated that pH, nutrient elements, *α* diversity, and network complexity significantly influenced network stability (*p* < 0.05; Supplementary Figure S6). The nutrient elements and network complexity directly enhanced network stability in the MP system, whereas α diversity exhibited the opposite effect. SEM analysis showed that pH negatively regulated α diversity in an indirect manner, which subsequently affected network stability (Figure 8A). By contrast, the soil pH and nutrient elements directly impacted network stability in a positive manner in the SPA system (Figure 8B). Moreover, pH negatively regulated nutrient elements in an indirect manner, thereby affecting network stability positively. According to the normalized total effect, pH and network complexity have positive effects on network stability in MP systems, while nutrient elements and *α* diversity have negative effects on network stability (Figure 8C). In SPA systems, pH, nutrient elements, α diversity, and network complexity all contribute positively to network stability (Figure 8D).

## 4 Discussion

### 4.1 Introduction of sanqi significantly increases *α* diversity but not abundance of NFB

Agroforestry systems serve as model systems for sustainable development and play a vital function in regulating tree growth (Tadesse et al., 2021), soil microbiomes (Wang et al., 2022), and soil nutrients (Manasa et al., 2024). Prior researches have verified that the introduction of plants can lead either to a substantial boost or no change in the abundance of NFB in the soils of agroforestry management systems (Beule and Karlovsky, 2021; do Rego Barros et al., 2021). However, the decrease in the abundance of NFB in the soil due to sanqi cultivation (*p* < 0.01) may be attributable to variations in plant species (Graungaard, 2015), root exudates (Strugstad and Despotovski, 2012; Na et al., 2018), and soil characteristics (Reed et al., 2011) across different agroforestry systems. Tillage has been reported to significantly affect the abundance and community structure of soil microorganisms (Hu et al., 2020); therefore, tillage was performed in both the systems to avoid any tillage-based effects on the NFB. The correlation analysis suggested that ST and TP were important factors that exhibited negative and positive correlation, respectively, with the abundance of NFB. Previous research has shown that the content of phosphorus may positively impact the abundance of NFB in the soil (Yang et al., 2019). Artificial pine forests are deficient in phosphorus (Fan et al., 2018), and the cultivation of sanqi further exacerbates the decrease in the soil phosphorus content, potentially resulting in a reduced abundance of NFB. In addition, the seasonal variations in SM and ST can affect the abundance of NFB in the soil (Mergel et al., 2001; Gao et al., 2021). We speculate that the highest abundance of NFB is found during autumn and winter, possibly due to the lower temperatures experienced during these seasons compared to spring and summer.

### 4.2 Sanqi cultivation alters the diversity, community structure, and composition of NFB

Interestingly, the cultivation of sanqi has been found to considerably improve the *α* diversity of NFB in the soil, which aligns with similar findings in other agroforestry systems such as the sugarcane–peanut and sugarcane–soybean systems (Liu et al., 2021; Pang et al., 2022). Sugars, amino acids, and hormones are released by plants introduced into the agroforestry systems, these can serve as additional substrates and energy sources for soil microbiomes, resulting in a rise in the α diversity of soil microbiomes (Zhang et al., 2004; Qian et al., 2017). The root exudates of sanqi primarily include sugars, saponins, flavonoids amino and organic acids (Liu et al., 2020), which may serve as the primary sources of carbon and nitrogen for NFB and lead to a further increase in α diversity. Moreover, the α diversity of NFB was the lowest in summer due to the seasonal regulation of energy input, availability and quality of the carbon source, and the community of NFB in the soil due to changes in ST and SM (Pereira et al., 2013; Che et al., 2018). Correlation analysis indicated that the α diversity of NFB was primarily influenced by SM and the contents of TK, TP, and NH_4_^+^–N. The potassium present in the soil may regulate the diversity of NFB by directly competing for NH_4_^+^–N-binding sites (Nieder et al., 2011) or indirectly by influencing nitrogen uptake by plants (Hou et al., 2019). Prior study has verified that NH_4_^+^–N exerts a detrimental effect on the α diversity of NFB (Herold et al., 2014). Therefore, the reduction in NH_4_^+^–N content in the soil along with the elevation in that of potassium and phosphorus after planting sanqi may be responsible for the increase in the α diversity of NFB.

The PERMANOVA analysis revealed that the system rather than the season significantly affected the community structure of NFB, which is inconsistent with a previous study on the cucumber-rapeseed system, wherein the community structure of NFB was found to be dramatically affected by sampling season rather than the treatment type (Gao et al., 2021). The variation may be attributable to the influence of plant species and seasonal dynamics on edaphic factors, which consequently impacts the composition of NFB (Huang et al., 2014; Hu et al., 2019). RDA analysis indicated that SM and the content of TK, TP, and NH_4_^+^–N notably affected the community structure of NFB as well. Sources of nitrogen play a regulatory role in the growth and reproduction of NFB (Mergel et al., 2001). Moreover, moisture may shape these NFB communities by regulating available nitrogen (Williams and Rice, 2007).

Intercropping has been demonstrated to influence the community composition of NFB in the soil (Gopal et al., 2021). For instance, the abundance of *Bradyrhizobium*, *Rhizobium*, and *Pseudomonas* significantly differed in the mulberry–alfalfa system compared to the monocropping mulberry system (Zhang et al., 2018), contributing to the enhancement of biological nitrogen fixation in the soil (VanInsberghe et al., 2015). Notably, the conversion from MP to SPA system triggered a noteworthy rise in the abundance of *Bradyrhizobium* and *Azospirillum*. *Bradyrhizobium* is recognized for its vital contribution to the N cycle in soil, particularly in nitrogen fixation in root nodules (VanInsberghe et al., 2015), while *Azospirillum* is recognized for its effective nitrogen-fixing capabilities (Miao et al., 2020). We hypothesize that the cultivation of sanqi may lead to an improvement in soil nutrient content and subsequently selective recruit certain microbial taxa involved in biological nitrogen fixation, thereby enhancing the plants’ ability to absorb and utilize nitrogen (Liu et al., 2021).

### 4.3 Sanqi cultivation enhances the ecological niche breadth and stochastic assembly of NFB

Ecological niche breadth is determined by the balance between intra- and inter-specific competition among microorganisms (Zhang and Okabe, 2020). In our research, the niche breadth of NFB in the soil was found to be wider in the SPA system compared to the MP system, which aligns with prior results reported by Li C. C. et al. (2022). The ecological niche breadth is inversely related to environmental stress (Jiao S. et al., 2022), which has been elucidated in the “ecological release” theory (Herrmann et al., 2021). The introduction of sanqi enhances the content of carbon and other nutrients (e.g., N and P) in the soil, alleviating the competition for resources among the NFB to facilitate their growth and reproduction (Gao et al., 2021), thereby expanding their niche breadth.

Deterministic and stochastic processes jointly shape the structure and functioning of the microbiome (Zhou and Ning, 2017). In alignment with the results of Li C. C. et al. (2022), the introduction of sanqi into the pine forests increased stochasticity in the community structure of NFB by reducing heterogeneous selection and increasing dispersal limitation while decreasing the prominent dependence on deterministic processes. This result is also supported by neutral models owing to the reduction in interspecific competition and increase in substrate availability (Jia et al., 2018). In conclusion, the cultivation of sanqi enhances the SOC content in the soil to facilitate the growth of NFB and reduces interspecific competition (Li C. C. et al., 2022), thereby enhancing the stochastic processes in establishing the community structure of NFB.

### 4.4 Sanqi cultivation increases the community complexity rather than stability of NFB

Microbial visualization networks can reveal the intricate interactions among species, offering an avenue for understanding the network complexity of microbiome (Faust and Raes, 2012). The network complexity of microbiomes can be measured using various topological characteristics (Xu et al., 2023). Compared to the MP system, the SPA system increased the network complexity but not the stability of NFB. The network complexity of microbiome regulates the network stability of the microbiome (Wagg et al., 2019), in fact, increased complexity was found to reduce network stability (Fan et al., 2018a), which contradicts the findings of this study. Network modularity is positively correlated with network stability, as this modular structure effectively isolates local perturbations, preventing their spread across the entire network and thereby maintaining overall stability (Li et al., 2023). Additionally, an increase in microbial cooperation tends to reduce network stability due to the high interdependence among species involved in cooperation. This interdependence can lead to a decline in other species when one species decreases, thus reducing stability (Coyte et al., 2015). In this study, both network modularity and microbial cooperation were observed to be higher in the SPA system compared to the MP system. This dual increase may counterbalance the respective effects on network stability, resulting in the network stability remaining unchanged. SEM analysis suggested that pH is a direct factor that increases the network complexity and stability of NFB in soil of the SPA systems. Previous studies revealed that the bacterial networks in an alpine grassland exhibited higher network complexity at a pH range of 5.17–8.92 (Chen et al., 2021). However, soil acidification may reduce the network stability of bacteria in desert grasslands (Liu et al., 2022). This difference arises because pH regulates the topological characteristics of bacterial networks by modifying both the community composition (Cho et al., 2019) and substrate availability (Pande and Kost, 2017). Therefore, the lower soil pH following the plantation of sanqi may indirectly impact the network complexity and stability of NFB by altering soil nutrient availability and diversity of NFB.

## 5 Conclusion

In the SPA system, Sanqi cultivation notably reduced the abundance of NFB and increased *α* diversity. The community structure of NFB exhibited notable disparities between the MP and SPA systems, with notable enrichment of *Bradyrhizobium* and *Azospirillum* in the SPA system. Moreover, the cultivation of sanqi broadened the ecological niche breadth of NFB and increased the stochasticity in community assembly (i.e., dispersal limitation) of NFB. Additionally, the SPA system increased the network complexity but not the stability of NFB. SEM analysis indicated that pH directly impacted the network complexity and stability of the NFB in the SPA system. To sum up, regulating the pH of the soil and supplementation with nitrogen can create a more favorable environment for cultivating sanqi.

## Data Availability

The datasets presented in this study can be found in online repositories. The names of the repository/repositories and accession number(s) can be found at: https://www.ncbi.nlm.nih.gov/, PRJNA1173392.
