# Supplementary material for: Introduction of Panax notoginseng into pine forests significantly enhances the diversity, stochastic processes, and network complexity of nitrogen-fixing bacteria in the soil
Source: Front Microbiol. 2025 Feb 3;16:1531875. doi: 10.3389/fmicb.2025.1531875 (PMC11830724; doi:10.3389/fmicb.2025.1531875)
Supplement: Supplementary file 1 [file Data_Sheet_1.docx]

Supplementary Material

**Supplementary Table 1.** Primers and reaction protocol for the *nifH* gene.

| Target gene | Primer sequences (5'-3') | Thermal profile (qPCR) | Thermal profile (PCR) |
| --- | --- | --- | --- |
| *nifH*-F*/nifH*-R  (Rosch et al. 2002) | F:5'-AAAGGYGGWATCGGY  AARTCCACCAC-3' | 95 °C for 3 min, followed by 35 cycles with 30 s at 95 °C, 30 s at 56 °C, 40 s at 72 °C, and finally 10 min at 72 °C. | 3 minutes at 95 °C, followed by 40 cycles with 30 s at 95 °C, 30 s at 60 °C, 45 s at 72 °C, and finally 10 min at 72 °C. |
|  | R: 5'-TTGTTSGCSG  CRTACATSGCCATCAT-3' |  |  |

**Reference**

Rosch, C., Mergel, A., and Bothe, H. (2002). Biodiversity of denitrifying and dinitrogen-fixing bacteria in an acid forest soil. Applied and environmental microbiology. 68, 3818-3829. doi: 10.1128/AEM.68.8.3818-3829.2002

**Supplementary Table 2.** Analysis of the average edaphic factors in the MP and SPA systems over the year.

| Soil varibles | Different systems | |
| --- | --- | --- |
|  | MP | SPA |
| ST (℃) | 11.68 ± 0.08 b | 11.88 ± 0.06 a |
| SM (%) | 21.25 ± 0.12 b | 26.18 ± 0.34 a |
| pH | 6.18 ± 0.00 a | 5.83 ± 0.01 b |
| BD (g/cm^-3^) | 0.92 ± 0.00 b | 1.01 ± 0.01 a |
| WFPS (%) | 31.08 ± 0.07 b | 43.09 ± 0.39 a |
| TK (g/kg^-1^) | 3.59 ± 0.00 b | 3.73 ± 0.00 a |
| TN (g/kg^-1^) | 0.91 ± 0.00 b | 0.94 ± 0.01 a |
| TP (g/kg^-1^) | 0.65 ± 0.00 a | 0.73 ± 0.00 b |
| NH_4_^+^-N (mg/kg^-1^) | 8.69 ± 0.09 a | 5.05 ± 0.07 b |
| NO_3_^-^-N (mg/kg^-1^) | 13.78 ± 0.09 b | 32.76 ± 0.07 a |
| SOC (g. kg^-1^) | 21.89 ± 0.24 b | 22.63 ± 0.18 a |

**Supplementary Table 3.** Seasonal fluctuations in edaphic properties within the MP and SPA systems.

|  | Soil varibles | Different seasons | | | |
| --- | --- | --- | --- | --- | --- |
|  |  | Autumn (October) | Winter (January) | Spring (April) | Summer (July) |
| MP | ST (℃) | 12.00 ± 0.00 c | 0.50 ± 0.00 d | 13.00 ± 0.00 b | 16.00 ± 0.00 a |
|  | SM (%) | 30.82 ± 1.13 a | 15.88 ± 0.40 c | 5.59 ± 0.85 d | 28.25 ± 0.97 b |
|  | pH | 5.69 ± 0.03 d | 6.35 ± 0.01 b | 6.46 ± 0.04 a | 6.17 ± 0.01 c |
|  | BD (g/cm^-3^) | 1.00 ± 0.04 a | 0.97 ± 0.04 ab | 0.90 ± 0.04 b | 0.94 ± 0.01 ab |
|  | WFPS (%) | 53.01 ± 0.78 a | 24.92 ± 1.32 c | 7.56 ± 0.67 d | 41.65 ± 1.11 b |
|  | TK (g/kg^-1^) | 3.62 ± 0.02 a | 3.59 ± 0.02 a | 3.34 ± 0.01 b | 3.38 ± 0.08 b |
|  | TN (g/kg^-1^) | 1.67 ± 0.02 a | 0.79 ± 0.01 d | 1.20 ± 0.04 b | 1.01 ± 0.02 c |
|  | TP (g/kg^-1^) | 1.08 ± 0.01 a | 0.77 ± 0.01 b | 0.64 ± 0.01 d | 0.67 ± 0.01 c |
|  | NH_4_^+^–N (mg/kg^-1^) | 3.67 ± 0.15 c | 14.42 ± 0.33 a | 2.25 ± 0.16 d | 4.54 ± 0.30 b |
|  | NO_3_^-^–N (mg/kg^-1^) | 32.17 ± 0.20 b | 6.62 ± 0.09 c | 34.45 ± 0.42 a | 6.28 ± 0.47 c |
|  | SOC (g/kg^-1^) | 22.47 ± 0.94 b | 13.02 ± 0.70 d | 32.27 ± 0.64 a | 15.36 ± 0.75 c |
| SPA | ST (℃) | 12.97 ± 0.46 B | 2.67 ± 0.29 D | 11.30 ± 0.17 C | 16.65 ± 0.30 A |
|  | SM (%) | 28.55 ± 1.22 A | 20.92 ± 0.19 B | 26.08 ± 0.89 A | 18.56 ± 1.40 B |
|  | pH | 5.71 ± 0.08 C | 6.01 ± 0.04 B | 6.32 ± 0.02 A | 5.55 ± 0.02 D |
|  | BD (g/cm^-3^) | 1.11 ± 0.01 AB | 1.16 ± 0.02 A | 0.89 ± 0.08 C | 0.99 ± 0.04 BC |
|  | WFPS (%) | 55.01 ± 1.50 A | 42.97 ± 1.65 B | 32.50 ± 0.45 C | 34.23 ± 2.79 C |
|  | TK (g/kg^-1^) | 3.68 ± 0.01 B | 3.77 ± 0.01 A | 3.57 ± 0.06 C | 3.73 ± 0.03 AB |
|  | TN (g/kg^-1^) | 0.57 ± 0.01 B | 1.06 ± 0.05 A | 1.03 ± 0.02 A | 0.48 ± 0.0 C |
|  | TP (g/kg^-1^) | 1.05 ± 0.01 A | 0.81 ± 0.00 B | 0.69 ± 0.01 C | 0.49 ± 0.01 D |
|  | NH_4_^+^–N (mg/kg^-1^) | 1.34 ± 0.09 C | 2.04 ± 0.06 B | 1.79 ± 0.19 B | 2.55 ± 0.09 A |
|  | NO_3_^-^–N (mg/kg^-1^) | 33.90 ± 0.41 B | 24.36 ± 0.34 C | 48.75 ± 0.57 A | 22.44 ± 0.35 D |
|  | SOC (g/kg^-1^) | 22.35 ± 0.72 B | 26.20 ± 0.43 A | 22.66 ± 0.44 B | 22.40 ± 1.03 B |

Note: different lowercase and uppercase letters represent significant differences between the MP and SPA systems over the four seasons, respectively.

**Supplementary Table 4.** PERMANOVA revealed the relative contributions of the system and season to the community structure of NFB.

| Factors | df | SS | MS | F.Model | Variation (R^2^) | Pr(>F) | Sig |
| --- | --- | --- | --- | --- | --- | --- | --- |
| System | 1 | 1.798 | 1.798 | 65.857 | 0.31 | 0.001 | ** |
| Season | 3 | 1.608 | 0.536 | 19.624 | 0.277 | 0.001 | ** |
| System × Season | 3 | 1.961 | 0.654 | 23.937 | 0.338 | 0.001 | ** |
| Residuals | 16 | 0.437 | 0.027 |  | 0.075 |  |  |
| Total | 23 | 5.804 |  |  | 1 |  |  |

**Supplementary Table 5.** The relative abundance of the top ten genera of NFB in the MP and SPA systems.

| Genus | Different systems | |
| --- | --- | --- |
|  | MP | SPA |
| *Unclassifed* | 47.91 ± 1.10 b | 84.26 ± 0.43 a |
| *norank_d__Bacteria* | 44.39 ± 0.93 a | 2.24 ± 0.25 b |
| *Bradyrhizobium* | 5.49 ± 0.90 b | 8.54 ± 0.60 a |
| *Paraburkholderia* | 0.82 ± 0.10 ns | 0.86 ± 0.21 ns |
| *Frankia* | 0.74 ± 0.39 ns | 0.76 ± 0.17 ns |
| *Azospirillum* | 0.05 ± 0.02 b | 1.13 ± 0.12 a |
| *Anaeromyxobacter* | 0.23 ± 0.15 b | 0.71 ± 0.15 a |
| *Beijerinckia* | 0.17 ± 0.01 b | 0.69 ± 0.16 a |
| *Sphingomonas* | 0.07 ± 0.04 ns | 0.12 ± 0.07 ns |
| *Xanthobacter* | 0 b | 0.19 ± 0.09 a |

**Supplementary Table 6.** Key OTUs of NFB network in the MP and SPA systems.

|  | OTU ID | Type | Phylum | Order | Degree |
| --- | --- | --- | --- | --- | --- |
| MP | OTU1217 | Connectors | unclassified_k__norank_d__Bacteria | unclassified_k__norank_d__Bacteria | 11 |
|  | OTU1227 | Connectors | Proteobacteria | Rhizobiales | 13 |
|  | OTU584 | Connectors | norank_d__Bacteria | norank_d__Bacteria | 16 |
| SPA | OTU1312 | Connectors | Proteobacteria | unclassified_p__Proteobacteria | 24 |
|  | OTU1332 | Connectors | Proteobacteria | Rhizobiales | 20 |
|  | OTU1373 | Connectors | Proteobacteria | Rhizobiales | 21 |
|  | OTU510 | Connectors | Proteobacteria | unclassified_p__Proteobacteria | 18 |
|  | OTU512 | Connectors | Proteobacteria | Rhizobiales | 18 |
|  | OTU532 | Connectors | unclassified_k__norank_d__Bacteria | unclassified_k__norank_d__Bacteria | 22 |
|  | OTU599 | Connectors | Proteobacteria | Burkholderiales | 24 |
|  | OTU766 | Connectors | Proteobacteria | Rhizobiales | 3 |


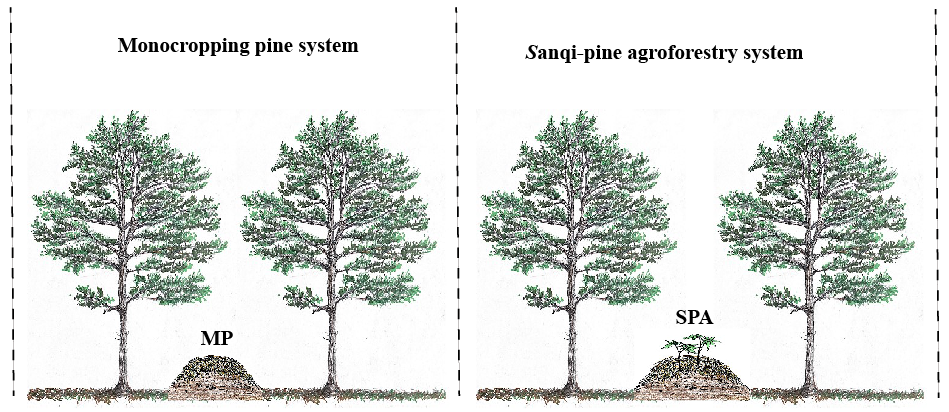


**Supplementary Figure 1.** Experimental design diagram.


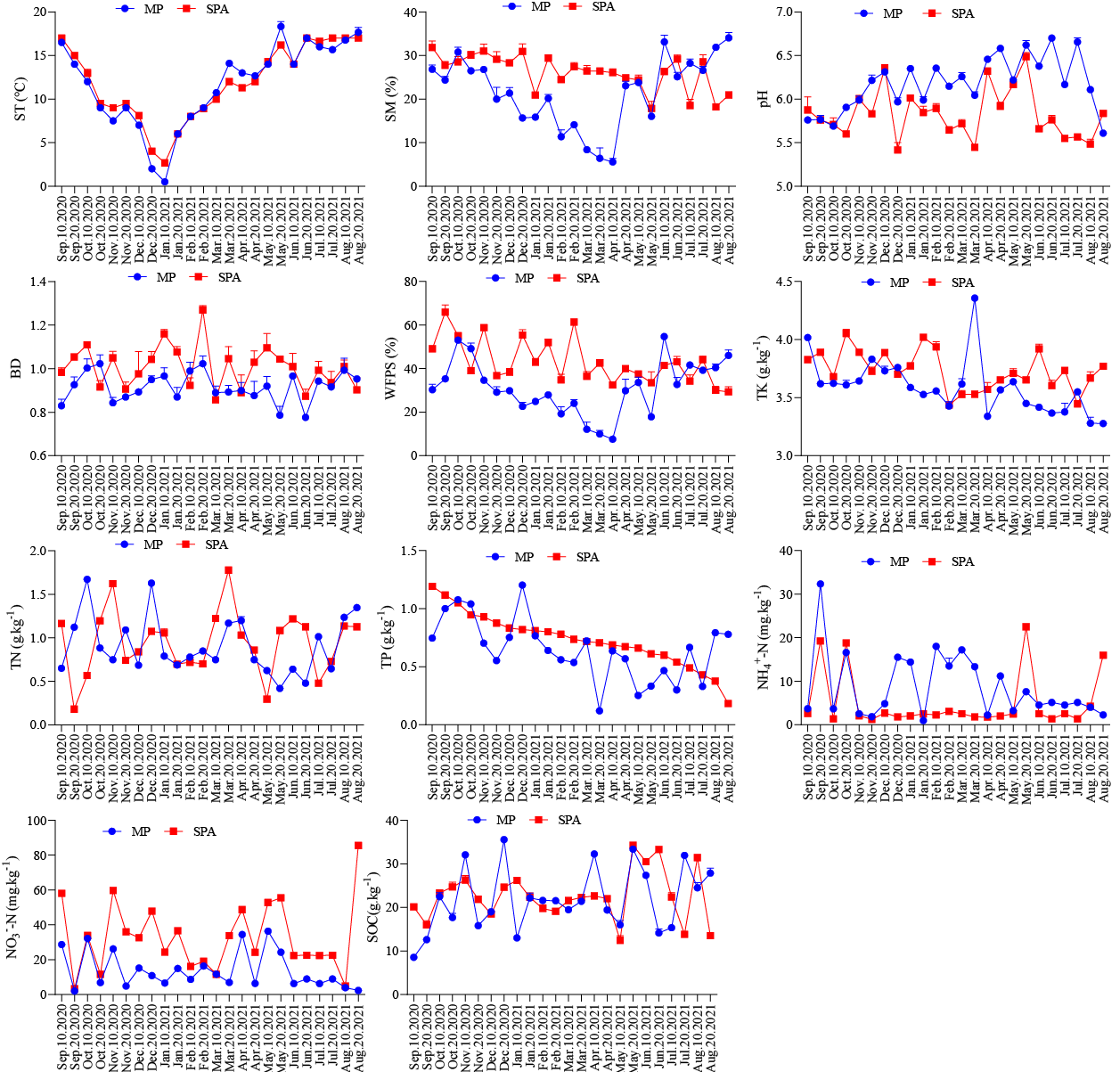


**Supplementary Figure 2.** Dynamic analysis of soil physicochemical properties in the MP and SPA systems over a year.


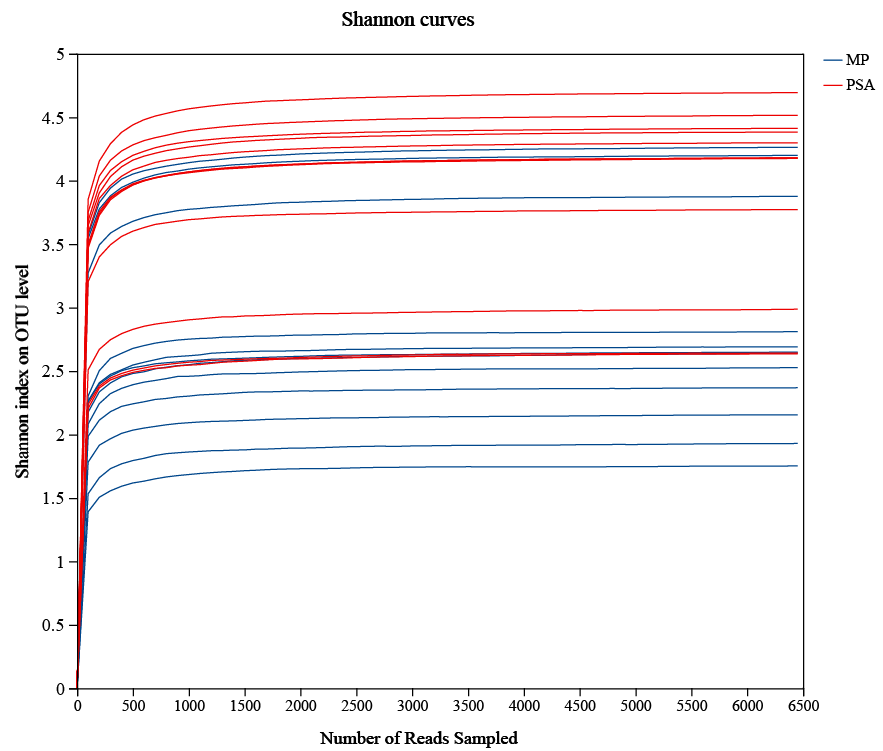


**Supplementary Figure 3.** Sparse curve analysis. The horizontal axis depicted the quantity of sequencing data randomly chosen, and the vertical axis denoted the observed Shannon diversity index.


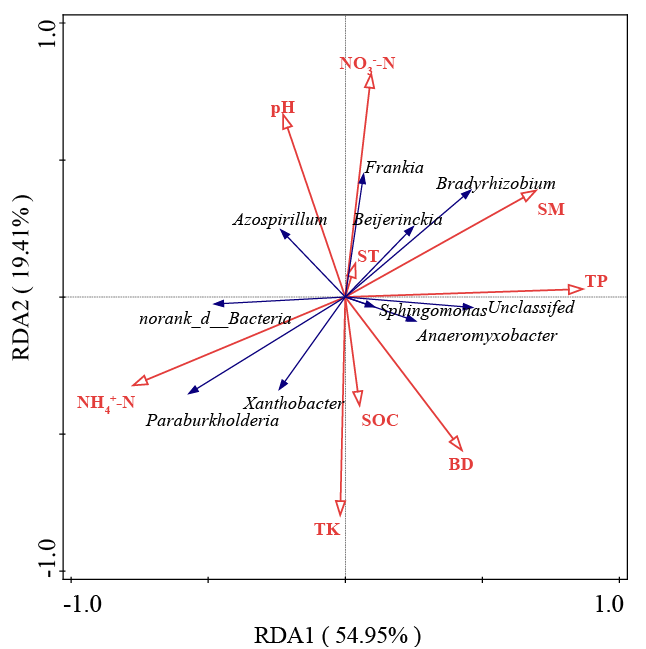


**Supplementary Figure 4.** RDA analysis of the correlation between the abundance of top ten genera of NFB and edaphic factors.


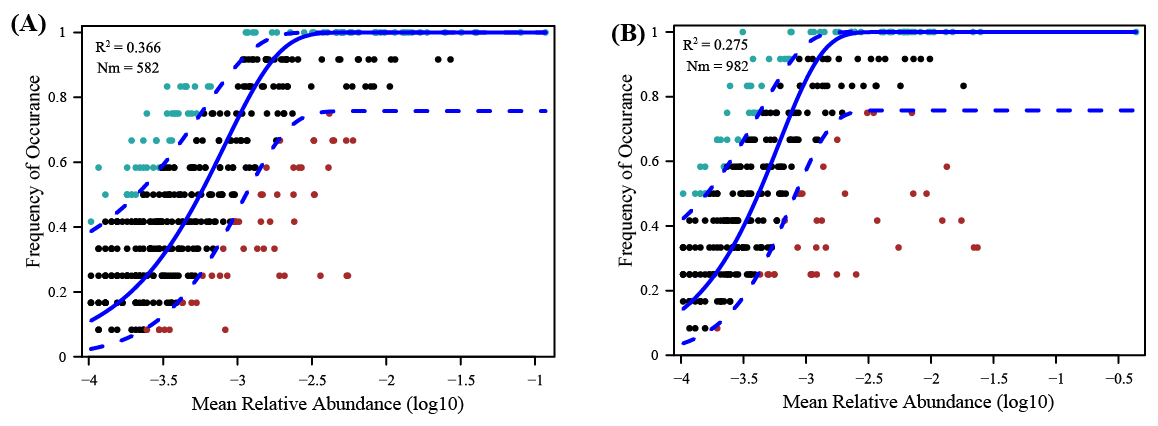


**Supplementary Figure 5.** Analysis of the community structure of NFB based on a neutral model. **A**, MP system, **B**, SPA system.


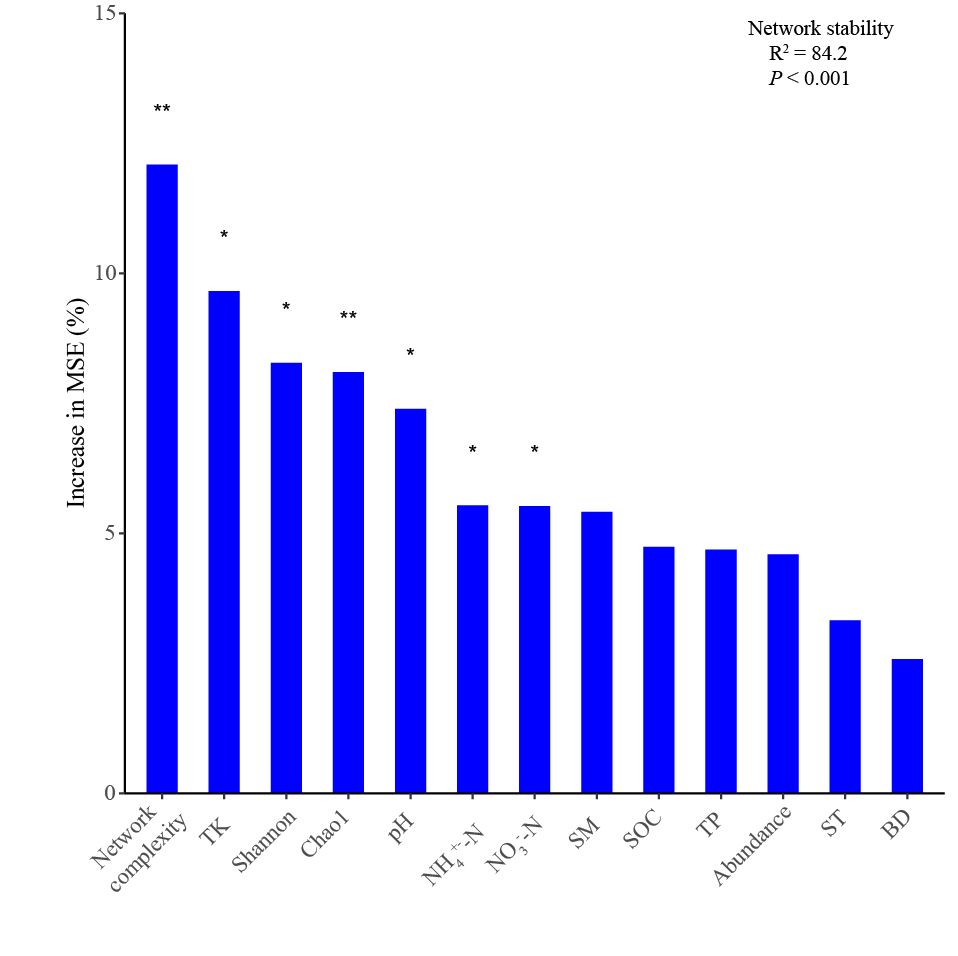


**Supplementary Figure 6.** Analysis of The random forest algorithm for the factors significantly affected the network stability of NFB.
